# Politics of disease control in Africa and the critical role of global health diplomacy: A systematic review

**DOI:** 10.34172/hpp.2021.04

**Published:** 2021-02-07

**Authors:** Vijay Kumar Chattu, W. Andy Knight, Anil Adisesh, Sanni Yaya, K. Srikanth Reddy, Erica Di Ruggiero, Obijiofor Aginam, Garry Aslanyan, Michael Clarke, M. Rashad Massoud, Ashish Jha

**Affiliations:** ^1^Department of Medicine, Faculty of Medicine, University of Toronto, Toronto, ON, Canada; ^2^Division of Occupational Medicine, St. Michael’s Hospital, Unity Health Toronto, Toronto, ON, Canada; ^3^Institute of International Relations, The University of the West Indies, St. Augustine, Trinidad and Tobago; ^4^Department of Political Science, Faculty of Arts, University of Alberta, Edmonton, Canada; ^5^School of International Development and Global Studies, Faculty of Social Sciences, University of Ottawa, Ottawa, Ontario, Canada.; ^6^The George Institute for Global Health, The University of Oxford, Oxford, UK; ^7^Bruyère Research Institute, University of Ottawa, Ottawa, Canada; ^8^WHO Collaborating Centre for Knowledge Translation and Health Technology Assessment in Health Equity, Ottawa, Canada; ^9^Centre for Global Health, Dalla Lana School of Public Health, University of Toronto, Toronto, ON, Canada; ^10^International Institute for Global Health, United Nations University, Kuala Lumpur, Malaysia; ^11^Special Programme for Research and Training in Tropical Diseases (TDR), World Health Organization (WHO), Geneva, Switzerland.; ^12^Schulich School of Medicine and Dentistry, Western University, London, Ontario, Canada; ^13^USAID Applying Science to Strengthen and Improve Systems (ASSIST) and University Research Co.,LLC, Center for Human Service, Maryland, USA; ^14^Harvard T.H. Chan School of Public Health and Harvard Global Health Institute, Cambridge, MA, USA; ^15^Brown University School of Public Health, Providence, RI, USA

**Keywords:** Politics, Disease control, Diplomacy, Africa, Noncommunicable diseases, communicable diseases, COVID-19, Global health, Health security, Epidemics

## Abstract

**Background:** Africa is facing the triple burden of communicable diseases, non-communicable diseases (NCDs), and nutritional disorders. Multilateral institutions, bilateral arrangements, and philanthropies have historically privileged economic development over health concerns. That focus has resulted in weak health systems and inadequate preparedness when there are outbreaks of diseases. This review aims to understand the politics of disease control in Africa and global health diplomacy’s (GHD’s) critical role.

**Methods:** A literature review was done in Medline/PubMed, Web of Science, Scopus, Embase, and Google scholar search engines. Keywords included MeSH and common terms related to the topics: "Politics," "disease control," "epidemics/ endemics," and "global health diplomacy" in the "African" context. The resources also included reports of World Health Organization, United Nations and resolutions of the World Health Assembly (WHA).

**Results:** African countries continue to struggle in their attempts to build health systems for disease control that are robust enough to tackle the frequent epidemics that plague the continent. The politics of disease control requires the crafting of cooperative partnerships to accommodate the divergent interests of multiple actors. Recent outbreaks of COVID-19 and Ebola had a significant impact on African economies. It is extremely important to prioritize health in the African development agendas. The African Union (AU) should leverage the momentum of the rise of GHD to (i) navigate the politics of global health governance in an interconnected world(ii) develop robust preparedness and disease response strategies to tackle emerging and reemerging disease epidemics in the region (iii) address the linkages between health and broader human security issues driven by climate change-induced food, water, and other insecurities (iv) mobilize resources and capacities to train health officials in the craft of diplomacy.

**Conclusion:** The AU, Regional Economic Communities (RECs), and African Centres for Disease Control should harmonize their plans and strategies and align them towards a common goal that integrates health in African development agendas. The AU must innovatively harness the practice and tools of GHD towards developing the necessary partnerships with relevant actors in the global health arena to achieve the health targets of the Sustainable Development Goals.

## Introduction

Africa is home to approximately 1.3 billion people. Africa’s estimated growth of Africa is estimated to be 3.4% for 2019 but is expected to grow up to 3.9% in 2020, and 4.1% in 2021.^1^ Extreme poverty remains persistently high in low-income countries in protracted conflicts, particularly in sub-Saharan Africa. Out of the 736 million people who lived on less than $1.90 a day in 2015, more than 413 million were from sub-Saharan Africa.^2^ According to the World Health Organization (WHO), Africa carries 25% of the world’s disease burden. Still, its share of global health expenditures is less than 1%, leaving more than half of its population without access to essential health services and manufactures less than 2% of the medicines consumed on the continent.^3^ Though the traditional communicable diseases such as HIV, tuberculosis (TB), and malaria have long been the most prominent contributors to the disease burden, sub-Saharan Africa has witnessed an epidemiological transition to non-communicable diseases (NCDs) in the last two decades.^4^

Infectious disease outbreaks in the region have become increasingly frequent and widespread over the past few decades. Although the epicenters of these outbreaks across the region are linked to differing socioeconomic contexts, the responses to these epidemics have been political and, at times, inherently troublesome for the marginalized groups. In common parlance, politics generally refers to “the activities that relate to influencing the actions and policies of a government or getting and keeping power in a government.”^5^ This definition has been critiqued as too narrow because it does not address the vast and multifaceted nature of the social activity in the realm of “politics.” Political scientists have observed over the years that because politics is generally about “who gets what, when and how”,^6^ it is, therefore, an activity inextricably linked to the phenomena of conflict and cooperation. In life, there are rival opinions, clashing needs and wants, opposing interests, and disagreements about the rules under which people live. So, conflict is inevitable in the political realm. Since everyone in society wants to influence the type of rules that govern social interactions, people would likely see it in their best interest to cooperate rather than being conflictual. Because of the divergent interests of diverse people within a society and the scarcity of available resources to meet basic needs, “politics is an inevitable feature of the human condition”.^7^ Therefore, this review aims to understand the politics of disease control in the African region and also to emphasize the critical role global health diplomacy (GHD) in addressing those challenges.

## Material and Methods

A systematic literature review was performed following the Preferred Reporting Items for Systematic Review and Meta-analysis (PRISMA) guidelines.^8^ A comprehensive literature search was conducted in all popular databases such as Medline/PubMed, Web of Science, Scopus, OVID, and Google Scholar search engines up to June 15, 2020. The review aimed to identify the studies on the “politics of disease control” and the “role of GHD” in the African context. A detailed search strategy using different combinations of MeSH (Medical Subjects Headings) keywords using Boolean operators “AND” and “OR” was conducted to identify the studies. The keywords used were: “Politics”, “disease control”, “epidemics/ endemics,” AND “global health diplomacy” in the African context. The resources also included WHO reports and resolutions of the World Health Assembly (WHA) and book publications.

**Figure 1:**
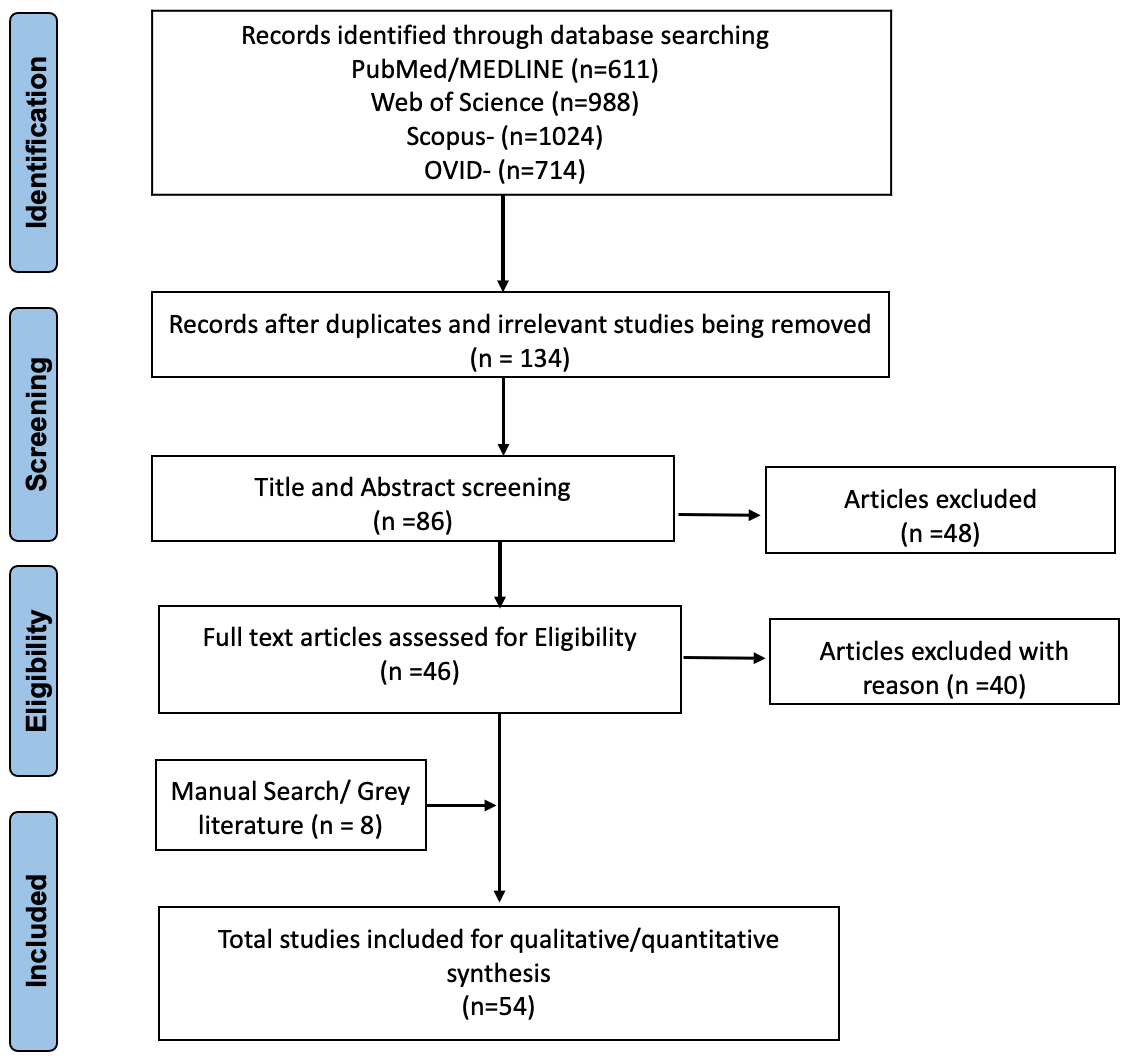
Flow chart showing the literature search strategy (as per PRISMA guidelines).

All the articles and reports published in English as full-peer reviewed manuscripts were screened for duplicates and potential missing studies. The grey literature included a few authentic reports from Regional and International Organizations such as the United Nations, WHO, African Union (AU), and African government websites that were also included to add more value to the information. To ensure quality, the document review process was independently conducted by two reviewers (VKC and KSR), and the identified studies were evaluated for suitability for this review. All the included studies in this study were scrutinized thoroughly, and the studies that met the selection criteria were retrieved for further analysis. Since there is a paucity of data available on this interdisciplinary area specific to the African context, we have identified many useful reports and collected information from important organizations’ webpages as mentioned above. Of the total 3337 results from the database search, we have identified 54 records in this review. The sources considered for this review are 28 journal articles, five books, 13 reports, and eight online sources of WHO, UN, AU, and African Population Health and Research Center. All the Annual Reports of the WHA for the past 13 years (2007-2019) were screened and reviewed to identify various initiatives and targets focused on the African region. These annual resolutions with targets for disease eradication, elimination, and prevention are studied carefully and categorized into three groups: communicable diseases, NCDs, and strengthening primary health care. The overall literature search strategy done as per PRISMA guidelines is shown below (Figure 1).

### Inclusion and exclusion criteria

Studies were eligible for inclusion if they were published in the English language, having the African context, discussing either infectious or chronic NCDs or health threats, politics of health, and disease control. Articles published in other than the English language and articles discussing other than African context were excluded.

In this review, which addresses the “politics of disease control” in Africa, we refer to politics as the extensive and diverse interactions of local, national, international, regional, bilateral and multilateral actors to influence communities’ health outcomes during infectious disease outbreaks in Africa. The study also considered the chronic NCDs and related health threats on the continent as constituents of the disease threats. As deployed in this paper, “politics” is the discourse of infectious disease epidemics caused by bacteria, viruses, and parasites, the strategies to contain the outbreaks, and the narratives assessing these strategies as reported in the literature.

## Results

The politics of disease control in Africa must be analyzed and understood in the context of the continent’s fragile health systems and the extent to which the tools of GHD have been deployed to address Africa’s region-specific health challenges. We propose a modified framework of the WHO’s health system building blocks with a broader macro-environment conducive to a region-specific sustainable and functional health system. Our focus is on three areas: (i) *Politics of Disease control in Africa*, (ii) *the rise of African Diplomacy for Disease control,* and (iii) *the role of GHD in strengthening Health Systems in Africa*. Along the way, we highlight several historical perspectives and unearth evidence that points to the politics of disease epidemics, accomplishments, and the challenges faced by the regional bodies, individual nations, and the fragile health systems in combatting epidemics. There is a need for continuous engagement of all relevant regional bodies in Africa and global stakeholders to conduct GHD for better health outcomes. GHD enables multiple stakeholders (both state and non-state actors) to improve the global health needs of humanity while also strengthening relations among state-society complexes.^9^

### Politics of disease control in Africa 

In the early 20^th^ century, news of the spread of the sleeping sickness epidemic around Lake Victoria became an international concern for British, Germans, French and Belgian scientists. The British laboratory at Entebbe, Uganda, generated research on its pathogen and vector but stayed largely out of the fray when developing disease control measures.The epidemic raised the alarm among German officials regarding their East African Empire. It resulted in the funding of an expedition by the famous bacteriologist Robert Koch from 1907 until World War I on Ssese islands, an archipelago of eighty-four islands in the northwestern part of Lake Victoria. Between 1901 and 1909, eight different expeditions sponsored by European governments and tropical medicine institutes fanned out across Africa to work on arsenic compounds (Atoxyl) and chemical dyes for the therapeutic experimental chemotherapies. E.g., the British scientists from the Liverpool School of Tropical Medicine at Entebbe used “Trypan red”, Arsenic and Atoxyl in Uganda, while the French sleeping sickness expedition in French Congo and the Belgian scientists in Belgian Congo used the Atoxyl without the certainty of these chemicals’ safety from animal experimentation.^10^ In the 21^st^ century, the African region continues to witness a high prevalence of infectious and parasitic diseases, respiratory infections, maternal and neonatal mortalities, and morbidities, nutritional deficiencies and NCDs have been the main causes of death in the African region for generations.^11^ In contrast to other regions of the world, with a burden of communicable or NCDs or both, Africa faces the challenge of a “Triple Burden (communicable and infectious diseases, NCDs, and nutritional conditions) which are discussed in this section.

### Communicable and vector-borne diseases 

The group of communicable or infectious diseases are caused by microorganisms such as bacteria, viruses, parasites, and fungi that can be spread, directly or indirectly, from one person to another. Though some are transmitted through bites from insects, others are also caused by ingesting contaminated food or water. Vector-borne diseases are human illnesses caused by parasites, bacteria, and viruses transmitted by vectors (Tables 1 and 2). The burden of these diseases is highest in Africa, being a tropical and subtropical region, and it disproportionately affects the poorest populations. Therefore, all the major outbreaks of communicable and infectious diseases have afflicted populations, claimed lives, and overwhelmed health systems, while neglected tropical diseases (NTDs) like chikungunya, leishmaniasis, and lymphatic filariasis have caused chronic suffering, life-long morbidity, disability, and occasional stigmatization. A recent review conducted by Fenollar and Mediannikov emphasized that the research on emerging infectious diseases needs to be identified as a priority.^12^ The 2014 Ebola outbreak was distinguished by the increased mobility within and between African countries after the end of civil wars in these countries (e.g., Liberia, Sierra Leone, Cote d’Ivoire), and also by the mobility of migrant workers due to the booming extractive mineral industry. These dynamics facilitated the transmission of the disease into capital cities and urban areas, where health infrastructure had not yet caught up with the growing population. In this context, another review conducted by Kapiriri and Ross on “politics of disease epidemics” highlighted that the responses to these outbreaks had been “political” and inherently burdensome especially to the marginalized populations.^13^ WHO was criticized for its ineffectiveness in handling the Ebola pandemic because of its response and poor coordination with local and regional actors. These inadequacies were partly the consequence of the shifting of priorities from infectious diseases to chronic NCD control, the result of which was inadequate funding for critical, rapid, and timely intervention to address emergent outbreaks in the region.^14^

### Chronic non-communicable diseases

According to the WHO’s estimates, the deaths from NCDs are likely to increase by 17% globally over the next ten years. By 2030, the African Region will experience a whooping 27% increase, that is 28 million additional deaths from these chronic conditions which are projected to exceed the combined deaths due to communicable, maternal, perinatal and nutrition-related diseases. In countries such as Mauritius, Namibia and Seychelles, over 50% of all reported adult deaths are caused by NCDs. This indicates that NCDs could soon be the leading cause of ill-health, disability, and premature death will hamper the socioeconomic development in the Africa Region.^36^ Some of the main NCDs ranked among the top 20 causes as per Global Health Estimates 2016 are highlighted below (Table 3).^37^

### Nutritional deficiencies and conditions

Of the total, 149 million children under 5 years of age globally, 22% of them were still chronically undernourished in 2018, and a big share of 36% was from sub-Saharan Africa. The Africa region has an estimated 209 million deaths caused by protein-energy malnutrition as per 2016 estimates (Table 3). According to the World Food Program 2019 report, in sub-Saharan Africa, the undernourished people increased from 195 million in 2014 to 237 million in 2017, and also has the highest prevalence of hunger increasing from 20.7 percent in 2014 to 23.2 percent in 2017.^38^ In sub-Saharan Africa, where two-thirds of the world’s maternal deaths occur, only 60 percent of births were assisted by skilled attendants. In 2017, though the total number of under-5 deaths dropped from 9.8 million in 2000 to 5.4 million, around 50 percent of those deaths were contributed by sub-Saharan Africa, indicating a great need to accelerate the continent’s progress.^2^

### The rise of African diplomacy for disease control 

**Figure 2:**
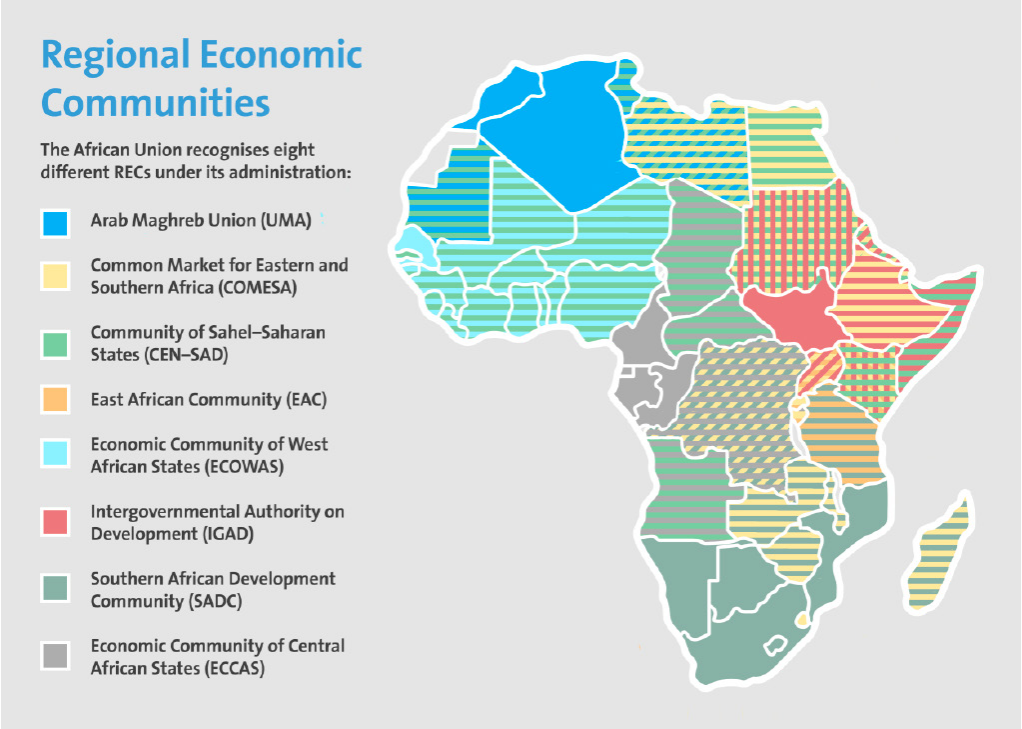
Regional Economic Communities of the Africa Union. Source: European Centre for Development Policy Management, (ECPDM) 2018.^40^

In May 2012, the 65th WHA approved an agreement between the Commission of the AU and the WHO. The AU, a continental union of 55 nation-states, was launched on July 9, 2002, in Durban, South Africa.^39^ The Secretariat of the AU Commission is based in Addis Ababa, Ethiopia. Although the African Regional Economic Communities (RECs) are primarily trading blocs that are, in some cases, given the mandate to pursue political and military cooperation towards achieving greater economic integration in the subregions, they are nonetheless described as the “building blocks” of the AU. Eight RECs are currently recognized by the AU (Figure 2). Many member countries have overlapping memberships and mandates, and sometimes competing activities place additional burdens on already over-stretched foreign affairs staff to attend various summits and meetings.

As per the Abuja Treaty, “the foundation of the African Economic Community (AEC) is the progressive integration of the activities of the RECs, with the establishment of full continental economic integration as the final objective towards which the activities of existing and future RECs must be geared”. The AEC envisages the creation of free trade areas, establishing customs unions, introducing a single market, a central bank, and a common currency. These ambitious plans aim to establish an economic and monetary union in six stages by the end of 2034.^41^ This development potentially serves the interests of the continent; however, they have to integrate strengthening the health systems to handle any type of regional/global epidemics as the movement of people in free borderless nations facilitate economy as well as infections.

AU has a total of 17 objectives, but only one of them addresses the health domain directly. Objective 14-“to work with relevant international partners in the eradication of preventable diseases and the promotion of good health on the continent”.^42^ This, in a way, indicates that health was not considered a top priority compared to other domains resulting in a lack of sufficient fund allocation at national levels. Similarly, of all the AU summits, only the Fifteenth AU Summit in Uganda in 2010 had a health theme on “Maternal, Infant, and Child Health and Development in Africa,” which again supports the notion that health has not been prioritized in the political agenda of the AU. Some of the major initiatives and political commitments made by African countries are listed below (Box 1).

The emergence of the Global Fund at the right time has revitalized the resources available for international attempts to control TB, malaria, and HIV in the African continent. Some scholars have argued that substantial reductions in overall child mortality and morbidity could be accomplished provided a small proportions of public health finance allocated toward the control of HIV/AIDS, TB, and malaria were re-directed toward the integrated control of neglected parasitic diseases.^43^ Molyneux et al argued that controlling Africa’s NTDs was one of “the most convincing ways to make poverty history” and therefore proposed the case for establishing a ‘new Global Fund’ on NTDs, which has not materialized, although significant resources are directed towards the control of NTDs.^44^

The “Global Vector Control Response (GVCR) 2017–2030” approved by the WHO in 2017, aims to provide strategic direction to nations and other development partners to strengthen vector control as an essential approach to disease prevention and outbreak response. This initiative was expected to be a game-changer for the African public health systems since it was intended to support the implementation of a comprehensive approach to vector control which would likely achieve not only the disease-specific national and global goals but also contributes to the attainment of health-related Sustainable Developmental Goals (SDGs) and universal health coverage (UHC).^45^

With the pandemic of COVID-19, the whole region’s economy and growth is greatly impacted by the lockdowns and other public health measures that significantly impact the economy. The virus has affected all 54 countries in with lockdowns implemented in over 30 countries. Hence the AU and the regional institutions need to refocus on the “Health is Wealth” strategy and have to reorient and prioritize health as in their policies to strengthen their fragile/ failed health systems in many countries. Many of the resolutions adopted by the WHA^46^ annually have impacted the African health policies and health systems (Table 4).

### Birth of African CDC and health research institutes

Before the Ebola epidemic, the African region had no continental Centers for Disease Control (CDC). There were regional Africa CDC hubs in Egypt, Nigeria, Gabon, Kenya, and Zambia, which handle high-risk viruses, health crises, research, and data collection. Some of these centers were built by the US. The role of the Africa CDC, launched in 2017 in the wake of the deadly 2014 Ebola crisis, is to respond to emerging and re-emerging disease outbreaks, including the current coronavirus. The African CDC is owned and part-funded by the AU’s 54 members, with additional funding support from the United States, China, the World Bank, Kuwait and Japan. Its secretariat is based at the AU headquarters in Addis Ababa. In 2015, the China-Africa Cooperation assured- “going forward hand in hand, cooperating with a win-win strategy, and developing with each other”—and pledged to provide USD 60 billion to support ten major cooperation plans across Africa.^47^ Moreover, the China-Africa health cooperation has supported the global health agenda on prevention, control, and elimination of some infectious diseases such as malaria, schistosomiasis, Ebola, TB, HIV/AIDS and NTDs. China’s health diplomacy also emphasized (1) the significance of health financing in establishing health development commitment, (2) investment in improving the gains and opportunities, and 93) value health priorities and planning.^48^ The African Population and Health Research Center, which emerged from a fellowship program of the Population Council in 1995, is a leading pan-African research institution based in Nairobi, Kenya that conducts high-quality policy-relevant research on various thematic areas such as population, health, education, urbanization, and other development issues across the continent.^49^

### The rising scope of GHD in Africa

GHD brings together the disciplines of public health, international affairs, management, law, and economics.^50^ The intersectoral linkages of health, foreign policy, security, and trade is central to GHD, and in recent years, the rising importance of health as a foreign policy concern is indicative of a renewed understanding among the diverse public and private actors within the global governance architecture on the need to make global health more visible on the agenda of multilateral and plurilateral organizations such as G7 and G20. Therefore, GHD ensures a serious commitment to health-related outcomes by governments, prioritizing context-specific health needs in securing bilateral/ multilateral support for addressing the critical macro level, non-medical interventions (outside the national health systems) to achieve the goals of UHC and health-related SDGs. For example, the Group of Twenty (G20) is an international forum, made up of 19 governments and Central Bank Governors plus the European Union (EU). The G20 members in their Leaders’ Declaration in 2019 made the commitment to move towards UHC through bolstering primary health care; granting greater access to medicines, vaccinations, nutrition, and water, and supporting proper sanitation, health promotion, and disease prevention measures; promoting healthy and active aging through the prevention of NCDs; improving emergency preparedness and response; and addressing antimicrobial resistance by identifying better models for antimicrobial drug research and development.^51^ The authentic and good examples of successful GHD are the International Health Regulations and the Framework Convention of Tobacco Control signed by all members of African states.

Dr. Matshidiso Moeti, the first female Director of the WHO’s Regional Office for Africa, had planned to achieve five priorities for Africa. They are “improving health security, strengthening national health systems, sustaining focus on the health-related SDGs, addressing the social determinants of health, and transforming the WHO secretariat in Africa into a responsive and results-driven organization.”^3^ Until now, Africa has been spending more on vertical disease control programs, undermining the importance of strengthening the existing health systems. Vertical approaches are usually disease-specific and support targeted clinical interventions delivered by a specialized service. Health experts have warned that spending funds disproportionately on disease-based initiatives in developing countries may compromise health systems and fragment complex interventions. By contrast, horizontal approaches tackle numerous interconnected health issues by reinforcing health systems and developing integrated delivery systems.^52^ Hence, we need a balance in Africa to implement the vital vertical initiatives which aim to decrease mortality quickly and also emphasize on general development that involves active intersectoral collaboration and comprehensive social initiatives.

**Figure 3:**
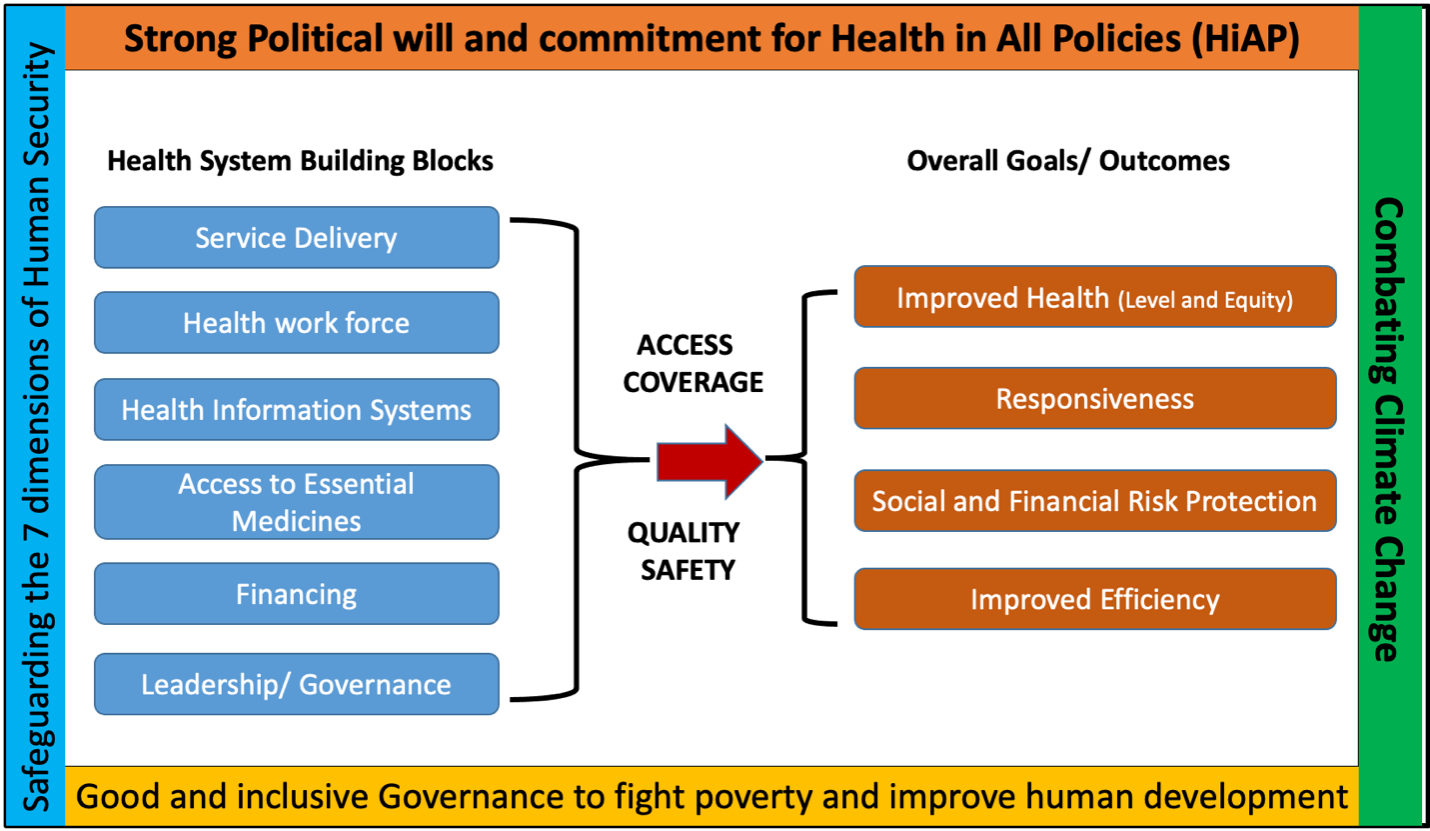
The essential four critical dimensions to reinforce the WHO’s health system building blocks. Source: Prepared by the authors

In addition to the six building blocks of health systems given by the WHO,^53^ it is critical to address and strengthen the broader dimensions outside the health systems, namely – (i) *The human security dimension*, (ii) *implementation of the Framework of Health in All Policies (HiAP),* (iii) *good governance to alleviate poverty and human development* and (iv) *combating climate change* (Figure 3). These macro-level factors outside the health sector provide the safeguards and pillars to build a resilient health system that responds to the burdens of emerging and re-emerging infectious diseases and NCDs. It was emphasized that the interactions between communicable and NCDs control offer broad implications for developing countries. E.g. activities in LMICs such as building up a clinical, laboratory, and regulatory capacity for handling both NCD and emerging disease threats can go a long way in improving health security locally and globally.^54,55^ Health in All Policies is “an approach to public policies across sectors that systematically takes into account the health implications of decisions, seeks synergies, and avoids harmful health impacts to improve population health”. Because of the many climate change-driven human security concerns, including food, water, and resource insecurities and constraints, which could ignite inter-group conflicts within the borders of nation-states, there is an urgent need to reorient the practice of GHD towards the development and sustainability of health systems.

Calling for more collaboration and political commitment by the largest economies, the WHO Director-General highlighted during the G20 summit in 2019 that “Health is a political choice and called on G20 countries to invest in health, a driver of jobs and growth, and in preparing for and preventing emergencies, rather than just responding to them” which has attracted a positive note from the summit but the statement still left much to be desired in terms of concrete goals and targets. Besides, he asked for support to fight against Ebola in the Democratic Republic of the Congo, to invest in global health preparedness now, before the next pandemic ravages the global economy, and commit to Health for All through UHC.^48^

## Box 1.

**Box 1.** Some of the major initiatives and political commitments made by African countries

- 2019 Joint statement by AU Commission, ALMA and the RBM Partnership to End Malaria: Africa’s leaders recommit to increase domestic resources to eliminate Malaria by 2030
- 2014 Addis Ababa commitment signed by 26 countries to increase domestic investment, strengthen NTD program goals, work towards global control and elimination targets, and strengthen overall health systems.
- 2014 Luanda Commitment to Universal Health Coverage in Africa.
- MDGs Africa Initiative, the Global Strategy for Women’s and Children’s Health, and the Harmonization for Health in Africa mechanism
- Adoption of the Brazzaville Commitment on Scaling up towards Universal Access to HIV and AIDS Prevention, Treatment, Care and Support by 2010
- 2008 Libreville Declaration on Health and Environment followed by the 2010 Luanda Commitment
- 2008 Algiers Declaration on strengthening research for health
- 2008 Ouagadougou Declaration on Primary Health Care and Health Systems in Africa
- declaration by African ministers of health of 2006 as Year of Acceleration of HIV Prevention in the Region
- adoption of Child Survival: A strategy for the African Region by the fifty-sixth session of the WHO Regional Committee for Africa in 2006
- 2005 WHO Regional Committee for Africa resolution on achieving the millennium development goals
- 2005 Maputo Declaration on Tuberculosis as an emergency
- 2004 resolution on the road map for accelerating the attainment of the MDGs related to maternal and newborn health in Africa
- 2001 Abuja Declaration requesting countries to allocate 15% of total public expenditure to the health sector

Source: Prepared by the authors based on the literature review.

## Strengths and Limitations

According to our knowledge, this systematic review is the first of its kind in this interdisciplinary area of politics, health, and diplomacy in the African context. Secondly, the review has taken into consideration all the published literature and the authentic grey literature from international organizations and global resolutions that have added great value and evidence to support the political commitments and the lacunae in implementation. Thirdly, the study explored the historical perspectives of the disease politics in Africa and highlighted the ‘Triple burden’ of diseases (epidemics of various communicable disease epidemics including the current COVID-19; the growing burden of NCDs and the Nutritional disorders). Finally, the review also identified that strengthening of health systems through multisectoral coordination is only possible through a strong political commitment.

Like any study, this review also has few limitations. Since there are not many studies done on this interdisciplinary aspect, and due to the paucity of the data from all nations in Africa, the evidence from many countries in Africa could not have been captured. As a literature review, we believe that many undocumented activities such as successful and failed strategies in some nations could not be discussed here, which would add value to the study. Though there are studies on epidemics of communicable diseases, most of them are focused on a few NTDs, and research publications covering all the domains of this interdisciplinary area are very few.

## Conclusion

The African region had made significant progress in recent years on the socioeconomic development of the continent. The AU needs to refocus and prioritize the continent’s health challenges by innovatively adapting the canons of GHD towards attracting more funding and developing collaborative partnerships with relevant actors in the global health domain. More than the vertical programs, the emphasis should be on HiAP as it addresses the health and health equity consequences of public policies on health systems and improves the accountability of policymakers. Developing a special cadre of global health diplomats would benefit the region with successful negotiations and -policymaking for disease control activities. GHD can be a game-changer for the region, especially during the COVID-19 pandemic crisis and post-COVID recovery efforts. Emphasizing the core issues of social determinants of health, gender-sensitive programs, prioritizing the endemic NTDs, addressing the challenges of climate change will reap benefits in the immediate as well as on a long-term basis. Strengthening the Health systems and earmarking specific funds for health from bilateral/ multilateral assistance could be done through the successful practice of GHD in the region. Upgrading health infrastructure and deploying available digital health solutions will improve access and strengthen the fragile health systems to achieve the UHC and related health targets of the SDGs in Africa.

## Funding

Nil.

## Competing interests

The authors declare that there is no conflict of interest.

## Ethical approval

Not Applicable.

## Authors’ contributions

Concept and design - VKC; definition of intellectual content -VKC, AK, KSR, OA, MC, OA; literature search - VKC; data acquisition - VKC, AA, KSR, EDR, GA; data analysis - VKC and KSR; initial manuscript draft preparation-VKC; manuscript editing and manuscript review -VKC, AK, AA, KSR, SY, EDR, GA, OA, MC, MR and AJ; Final editing - VKC. All authors have accepted the final version of the manuscript.

## Disclaimer

The views expressed in this publication are those of the authors based on their experience and the available evidence. The authors disclaim any responsibility for any damage incurred as a consequence of the use or application of the content of the article. Further the authors claim that no part of this paper is copied from other sources.

**Table 1:** Emerging and re-emerging viral diseases in the African continent (main outbreaks since 2000 until the present)

**Name of virus**	**Countries affected (year) and implications**	**Response by WHO and** **interventions by government**	**References**
Novel coronavirus 2019 (COVID-19)	• The first confirmed case was in Egypt on February 14, 2020, and the first case in sub-Saharan Africa was in Nigeria. • Over 200 countries have been affected gradually with a total of 95 million cases and 2.1 million deaths as of January 20, 2021.	On January 30, 2020, the WHO had declared as Public Health Emergency of International Concern (PHEIC). On March 11, 2020, the WHO declared COVID-19 a pandemic. Many countries have implemented lockdown, the closure of borders; implementing a 14-day quarantine, social distancing, the wearing of masks, and other public measures.	^15-17^
Zika virus	• First outbreak detected: Cabo Verde (2015) • African Region had autochthonous mosquito-borne transmission.	Declared PHEIC on February 8, 2016 Vector-control measures	^18^
Ebola virus	• Guinea, Sierra Leone, Liberia, Mali, Nigeria- largest outbreak, 28646 cases including 11 323 deaths • Epidemic cost a total of USD 4.3 billion, loss of human resources, including health care staff, food security issues, and decrease in cross-border trade. • North Kivu province (eastern DRC), August 1, 2018—ongoing; as of August 25, 2018, 79 confirmed cases, 42 deaths • DRC 2018: Équateur province (northwest of DRC), May 8 –25 July 2018: 38 confirmed cases, 17 deaths; 7 cases were healthcare workers, 2 of whom died • DRC (2007, 2008–2009, 2012, 2014, 2017); Uganda (2007) • Gabon and Republic of the Congo (2001–2003)	Declared PHEIC on August 8, 2014	^19-21^
Yellow fever	• Nigeria (2017; Uganda (2016) • Angola and DRC (2015–2016): 7334 suspected cases, 962 cases confirmed (393 deaths) • DRC (2014); Chad, DRC, Ethiopia (2013) • Cameroon, Republic of South Sudan (2012–2013) • Ghana (2012); Côte d’Ivoire, Sénégal, Sierra Leone, Uganda (2011); Côte d’Ivoire, DRC, Uganda (2010) • Central African Republic, Guinea, Sierra Leone (2009) • Burkina Faso, Central African Republic, Guinea, Liberia (2008); Côte d’Ivoire, Senegal, Togo (2006) • Burkina Faso, Côte d’Ivoire, Guinea, Republic of South Sudan, Senegal (2005); Burkina Faso, Liberia, Mali (2004); Ghana, Guinea, South Sudan, Sierra Leone (2003); Senegal (2002–2003); Côte d’Ivoire (2001–2003) • Guinea, Liberia (2000–2001); Nigeria (2000)	Sustained vaccination in human population	^22-24^
Rift Valley fever	• Republic of Mauritania (2016) • Republic of Niger (2016) • Republic of South Africa (2010) • Madagascar (2008–2009) • Sudan and Tanzania (2007) • Kenya and Somalia (2006–2007) • Egypt (2003)	Sustained vaccination in animals; vector-control measures	^25,26^
Monkey Pox	• Nigeria (2017): 146 suspected cases and 42 laboratory-confirmed cases, with the death of confirmed monkeypox patient with a history of immunosuppression • Central African Republic (2015–2016): 13 cases, fatality rate of 67% among children aged <10 years	Reduce the risk of wildlife-to-human transmission	^27,28^
Measles	• Measles outbreaks occur every year throughout Africa • DRC (2010>–2013): largest outbreak, 294 455 cases, 5045 deaths	Sustained vaccination in human Population	^29^
Chikungunya virus	• DRC (2011) • Several outbreaks in Guinea, Tanzania, Sudan, Gabon, and Cameroon (2004–2007) • Kenya (2004): largest outbreak (almost half a million)	Vector-control measures	^30^
H1N1 influenza	• Affected 21 African Nations	Declared (PHEIC) on April 25, 2009 Declared a global pandemic on July 1, 2009 Declared as post-pandemic on August 10, 2010	^31^

Source: Prepared by the authors and modified from Fenollar and Mediannikov.^12^

**Table 2:** Major emerging and re-emerging bacterial disease outbreaks in the African continent (since 2000 )

**Name of bacterial**	**Countries affected (year)**	**Implications**	**Response by WHO and/** **by government**	**References**
Vibrio cholera	Algeria (2018) DRC, Mozambique, Somalia (2017–2018) Kenya, Zambia (2017) United Republic of Tanzania (2015–2018) Republic of South Sudan (2014) Central Africa, DRC, Sierra Leone, Republic of the Congo (2012) Zimbabwe (2008–2009) Zimbabwe (2011) West Africa (2008) Angola and the Republic of South Sudan (2006) West Africa (2005) Niger (2004–2005) Cameroon, Chad, Zambia (2004) Benin, Côte d’Ivoire, DRC, Liberia, Mali, South Africa, Uganda, Zambia (2003) Mozambique (2002–2004): Burundi, Côte d’Ivoire, DRC, Liberia, Malawi, Niger (2002) Chad, Nigeria, Tanzania, West Africa (2001) South Africa (2000–2001) Madagascar, Somalia (2000)	Since mid-August 2018, 41 confirmed cases, two deaths. 33 421 cases, 542 deaths DRC (2015) Largest outbreak, 98 585 cases, 4000 deaths 17 265 cases, 102 deaths	Appropriate water and sanitation facilities Oral cholera vaccination (transient protection about 3–5 years) To be alert during conflicts or natural disasters	^32,33^
Yersinia pestis	Madagascar (2018): Madagascar (2017): DRC (2018): DRC (2005, 2006) Algeria, DRC, Mozambique, Uganda (2003) DRC, Malawi, Mozambique, Uganda, United Republic of Tanzania (2002) DRC, Uganda, United Republic of Tanzania (2001) Recurrent outbreaks in Madagascar, DRC, Uganda, United Republic of Tanzania (2000)	104 cases, 34 deaths One of the worst outbreaks in the world in the past half-century, 661 cases 87 deaths 133 cases and five deaths	Reduce the risk of wildlife-to-human transmission	^34,35^

Source: Prepared by the authors and modified from Fenollar and Mediannikov.^12^

**Table 3:** Estimated deaths by cause for WHO Africa Region

**Rank**	**Cause**	**Deaths (000s )**	**% of total deaths**	**Cumulative % of total deaths**	**CDR (per 100 000 population)**
0	All Causes	8845	100.0	100.0	867.2
1	Lower respiratory infections	917	10.4	10.4	89.9
2	HIV/AIDS	719	8.1	18.5	70.5
3	Diarrhoeal diseases	653	7.4	25.9	64.0
4	Ischaemic heart disease	512	5.8	31.7	50.2
5	Malaria	408	4.6	36.3	40.0
6	Tuberculosis	405	4.6	40.9	39.8
7	Stroke	373	4.2	45.1	36.6
8	Preterm birth complications	344	3.9	49.0	33.7
9	Birth asphyxia and birth trauma	323	3.7	52.6	31.7
10	Road injury	284	3.2	55.8	27.8
11	Protein-energy malnutrition	209	2.4	58.2	20.5
12	Maternal conditions	194	2.2	60.4	19.0
13	Congenital anomalies	189	2.1	62.5	18.6
14	Meningitis	186	2.1	64.6	18.2
15	Cirrhosis of the liver	174	2.0	66.6	17.1
16	Neonatal sepsis and infections	173	2.0	68.5	16.9
17	Diabetes mellitus	168	1.9	70.4	16.4
18	Chronic obstructive pulmonary disease	118	1.3	71.8	11.5
19	Interpersonal violence	106	1.2	73.0	10.4
20	Alzheimer disease and other dementias	91	1.0	74.0	8.9

Source: WHO-Global Health Estimates 2016.

**Table 4:** Global strategies adopted by the WHA impacting the disease control policies of African countries

**Year**	**Communicable diseases**	**Non-communicable diseases**	**Primary health care, nutrition and WASH**
2019	**WHA72.5** Antimicrobial resistance	**WHA72.16** Emergency care systems for universal health coverage: ensuring timely care for the acutely ill and injured	**WHA72.2** Primary health care **WHA72.8** Improving the transparency of markets for medicines, vaccines, and other health products; **WHA72.6** Global action on patient safety; **WHA72.7** Water, sanitation and hygiene (WASH) in health care facilities
2018	**WHA71.4** Cholera prevention and control	**WHA71.2** High-level Meeting of the General Assembly on the Prevention and Control of NCDs; **WHA71.6** WHO’s global action plan on physical activity 2018–2030	**WHA71.7** Digital health **WHA71.9** Infant and young child feeding
2017	**WHA70.16** The “Global Vector Control Response (GVCR) 2017–2030; **WHA70(10**) Review of the Pandemic Influenza Preparedness Framework; **WHA70(11**) Implementation of the IHRs (2005)	**WHA70.12** Cancer prevention and control in the context of an integrated approach **WHA70(17)** Global action plan on the public health response to dementia1	**WHA70.14** Strengthening immunization to achieve the goals of the global vaccine action plan
2016	**WHA69.11** Health in the 2030 Agenda for Sustainable Development	**WHA69.3** Global strategy and action plan on ageing and health 2016–2020; **WHA69.5** WHO global plan of action to strengthen the role of the health system within a national multisectoral response to address interpersonal violence, in particular against women and girls, and against children	**WHA69.8** United Nations Decade of Action on Nutrition (2016–2025) **WHA69.9** Ending inappropriate promotion of foods for infants and young child **WHA69.10** Framework of Engagement with Non-State Actors
2015	**WHA68.2** Global technical strategy and targets for malaria 2016–2030**WHA68.3** Poliomyelitis; **WHA68.4** Yellow fever risk mapping and recommended vaccination for travellers; **WHA68.6** Global vaccine action plan; **WHA68.7** Global action plan on antimicrobial resistance	**WHA68.8** Health and the environment: addressing the health impact of air pollution	**WHA68.19** on infant and young child nutrition, appropriate feeding practices **WHA68.6** Global vaccine action plan **WHA68.9** Framework of engagement with non-State actors
2014	**WHA67.1** Global strategy and targets for tuberculosis prevention, care, and control after 2015; **WHA67.6** Viral hepatitis	**WHA 67** Contributing to social and economic development: sustainable action across sectors to improve health and health equity	**WHA67.7** WHO global disability action plan 2014–2021: better health for all people with disability
2013	**WHA66.12** Neglected tropical diseases (including the Global Plan to Combat NTDs 2008–2015)	**WHA66.8** Comprehensive mental health action plan 2013–2020; **WHA66.10** Follow-up to the Political Declaration of the High-level Meeting on NCDs	**WHA66.9** Disability
2012	**WHA65.17** Global vaccine action plan **WHA65.5** Poliomyelitis: intensification of the global eradication initiative; **WHA65.18** World Immunization Week; **WHA65.21** Elimination of schistosomiasis; **WHA65.23** Implementation of the IHRs (2005)	**WHA65.3** Strengthening non-communicable disease policies to promote active ageing **WHA65.4** The global burden of mental disorders and the need for a comprehensive, coordinated response from health and social sectors at the country level	**WHA65.6** Comprehensive implementation plan on maternal, infant, and young child nutrition
2011	**WHA64.1** Implementation of the IHRs (2005); **WHA64.5** Pandemic influenza preparedness: sharing of influenza viruses and access to vaccines and other benefits; **WHA64.14** Global health sector strategy on HIV/AIDS,2011–2015**WHA64.15** Cholera: a mechanism for control and prevention; **WHA64.16** Eradication of dracunculiasis; **WHA64.17** Malaria; **WHA64(11)** Smallpox eradication: destruction of variola virus stocks	**WHA64.11** Preparations for the High-level Meeting of the United Nations General Assembly on the Prevention and Control of Noncommunicable Diseases **WHA64.27** Child injury prevention **WHA64.28** Youth and health risks	**WHA64.6** Health workforce strengthening **WHA64.7** Strengthening nursing and midwifery **WHA64.9** Sustainable health financing structures and universal coverage **WHA64.13** Working towards the reduction of perinatal and neonatal mortality **WHA64.24** Drinking-water, sanitation, and health
2010	**WHA63.1** Pandemic influenza preparedness: sharing of influenza viruses and access to vaccines and other benefits; **WHA63.18** Viral hepatitis; **WHA63.19** WHO HIV/AIDS strategy for 2011–2015; **WHA63.20** Chagas disease: control and elimination	**WHA63.13** Global strategy to reduce the harmful use of alcohol **WHA63.25** Improvement of health through safe and environmentally sound waste management	**WHA63.3** Advancing food safety initiatives **WHA63.17** Birth defects **WHA63.23** Infant and young child nutrition
2009	**WHA62.15** Prevention and control of multidrug-resistant tuberculosis and extensively drug-resistant tuberculosis	**WHA 62.14** Reducing health inequities through action on the social determinants of health	**WHA62.12** Primary health care, including health system strengthening; **WHA62.13** Traditional medicine
2008	**WHA61.2** Implementation of the IHRs (2005); **WHA61.15** Global immunization strategy	**WHA61.14** Prevention and control of NCDs: implementation of the global strategy; **WHA61.4** Strategies to reduce the harmful use of alcohol; **WHA61.19** Climate change and health	**WHA61.16** Female genital mutilation **WHA61.20** Infant and young child nutrition: biennial progress report
2007	**WHA60.14** Poliomyelitis: mechanism for management of potential risks to eradication; **WHA60.18** Malaria, including a proposal for the establishment of World Malaria Day; **WHA60.19** Tuberculosis control: progress and long-term planning	**WHA60.17** Oral health: an action plan for promotion and integrated disease prevention **WHA60.21** Sustaining the elimination of iodine deficiency disorders; **WHA60.23** Prevention and control of NCDs: implementation of the global strategy; **WHA60.24** Health promotion in a globalized world; **WHA60.26** Workers’ health: global plan of action	**WHA60.16** Progress in the rational use of medicines **WHA60.20** Better medicines for children

Source: Prepared by the authors based on the WHA Annual Reports.
